# Adolescents’ Experience of Stress: A Focus Group Interview Study with 16–19-Year-Old Students during the COVID-19 Pandemic

**DOI:** 10.3390/ijerph19159114

**Published:** 2022-07-26

**Authors:** Camilla Perming, Åsa Thurn, Pernilla Garmy, Eva-Lena Einberg

**Affiliations:** 1 Department of Nursing and Health Sciences, Faculty of Health Sciences, Kristianstad University, SE-291 88 Kristianstad, Sweden; camillaperming@hotmail.com (C.P.); asa.thurn@hastveda.net (Å.T.); evalena.einberg@hkr.se (E.-L.E.); 2 Department of Health Sciences, Faculty of Medicine, Lund University, SE-221 00 Lund, Sweden

**Keywords:** adolescents, stress, experience, focus group interviews, qualitative study

## Abstract

The purpose was to investigate stress among adolescents (16–19 years old) during the coronavirus disease 2019 (COVID-19) pandemic. Focus group (n = 9) interviews with students at upper secondary schools (16–19 years old, n = 41) were conducted in southern Sweden during the COVID-19 pandemic in 2021. The interviews were analyzed by qualitative content analysis. The analysis revealed five categories of experience of stress: (1) School-related stress, (2) Stress in relationships and leisure, (3) How stress manifests itself, (4) Stress can increase performance, and (5) Experiences of counteracting stress. The adolescents in the study experienced stress linked to school and relationships, partly due to the COVID-19 pandemic. Distance education during the COVID-19 pandemic increased stress levels. The adolescents stated that high stress levels were experienced negatively and impaired their ability to perform, while moderate stress could contribute to achieving goals and improving performance. School nurses and other health professionals who meet with adolescents are important to support adolescents experiencing stress.

## 1. Introduction

Stress in adolescents is a growing problem. Perceived stress at school has increased sharply, especially among girls [1]. Since 1993, school stress in 15-year-olds has doubled in both boys and girls [1]. According to the World Health Organization (WHO) [2], adolescence falls between the ages of 10 and 19 years, during which adolescents develop physically, mentally, and socially. The body is exposed to hormonal stress and child-to-adult changes; they develop their independence and self-esteem, which means that they become sensitive to the opinions of others [2]. During late adolescence, which occurs at 16–19 years old [2], most young people in Sweden go to upper secondary school. The start of the coronavirus disease 2019 (COVID-19) pandemic in 2020 affected upper secondary schools with a rapid switch from education on-site to distance education, which increased upper secondary school students’ experiences of stress [3]. Understanding adolescents’ perspectives is important in developing preventive work in school health services. In earlier studies, experiences of stress in early and middle adolescence have been investigated [4,5]. However, little is known about the experience of stress in late adolescence. Therefore, in this study, we examined experiences of stress among students aged 16–19 at upper secondary schools during the COVID-19 pandemic in southern Sweden.

Hans Seyle [6] describes stress as the body’s reaction to physical and mental stress. Stress is a natural part of life that can occur in several different situations, as intense as a painful blow or a passionate kiss. Stress is associated with negative symptoms, such as fatigue and exhaustion, but stress can also act as a driving force that increases performance. Unhealthy stress can cause symptoms such as fatigue, diffuse pain, stomach problems, weight loss or weight gain, and decreased appetite. Prolonged stress can lead to somatic illness and mental illness [6]. A Swedish longitudinal study by Högberg et al. [7], which examined the development of stress in adolescents <24 years old from 1993 to 2017, confirms that stress has increased in adolescents. The largest increase occurred after 2009 and was highest in girls. The study also showed a sharp increase in psychosomatic symptoms [7].

Several factors affect stress in adolescents. The family’s influence was greatly important for stress and inner abilities such as how adolescents handle emotions, conflicts, and events in life [8]. Additional factors were school-related stress. School-related stress was linked to demands on one’s performance, grading, and conflicts with teachers [8,9,10]. Stress was also linked to relationships. This involves romantic relationships and friendships, where conflicts in the relationships cause stress [8]. Perceived academic demands are risk factors for mental ill-health in adolescence [11]. Studies have shown that stress levels are high among students in high-resource areas, where there is pressure to achieve the best possible grades [12,13]. A focus group study among Swedish students (13–15 years old) during the COVID-19 pandemic found that students worried about the pandemic [5]. There is a bidirectional relationship between stress and sleep difficulties. Sleep deprivation reduces the ability to cope with stress, and stress in turn causes sleep problems [14]. Girls are more affected by sleep difficulties and stress than boys, and girls are more likely to use technology when going to bed [14]. The use of digital media and mobile phones has increased sharply among adolescents [15]. Adolescents today live with digital stress that can be divided into four components: accessibility, fear of missing out, social acceptance, and media congestion [15]. Being available 24 h a day is a stress factor that makes it difficult to relax. Fear of missing out creates a compulsion to use and be updated on social media to keep up with social interactions. The need for social acceptance creates stress and anxiety about comments about how others react to posts. Media congestion is linked to the number of impressions and stimuli from digital media, giving less rest and recovery time [15]. 

A well-known theory is the salutogenic model “Sense of Coherence”, SOC, by Aaron Antonovsky [16]. Sense of coherence promotes health and increases resilience to stress [17]. In SOC, manageability, comprehensibility, and meaningfulness are used as central concepts [16]. SOC is important for how adolescents handle stress [18]. Support from classmates and teachers also has a direct positive impact on students’ health and stress management. A supportive school environment contributes to a reduction in school-related stress and increases SOC. Family support plays a significant role in adolescents’ SOC, and well-being as a positive family dynamic with good relations with the parents increases security [19]. A Norwegian cross-sectional study showed that boys felt happier in life than girls, who rated higher on all stressors; the largest differences between the sexes were seen in stress related to school results, peer pressure, and conflicts [20]. Overall stressors that emerged included home life, school-related stress, and conflicts in relationships. The relationship between belief in one’s ability and stress was investigated in adolescents and supports the theory that believing in one’s ability increases the joy of life and, in turn, gives a better ability to handle stress. Furthermore, in another study on the connections between school stress, the joy of life, and depressive symptoms, it was also found that the children who estimated higher joy in life better handled stress related to schoolwork [21]. 

The COVID-19 pandemic greatly impacted the world from 2020 to 2021, with students having distance education. Sweden chose a milder way of lockdown than many other countries. Students aged 16–19 had distance education during the pandemic but people were, for example, always allowed to walk outdoors [22]. School nurses found that adolescents were concerned about spreading infection, becoming infected themselves, their academic performances, and longing for socialization [23]. School nurses tried to alleviate stress among adolescents through being easily assessable and providing tools for stress management [24]. 

It is important to investigate the experience of stress among adolescents during this period. The study aimed to investigate the stress experience among adolescents (16–19 years old) during the COVID-19 pandemic in Sweden. 

## 2. Materials and Methods

The study was conducted as a qualitative study with interviews in focus groups. The study has been approved by the Swedish Ethical Review Authority (2018/842). The COREQ guidelines were used [25].

### 2.1. Data Collection

Purposive sampling was used. The school administrations in three municipalities with six upper secondary schools in southern Sweden were informed about the study during spring 2021, and two schools from two municipalities accepted to participate. The schools had 350–450 students each and provided both university preparatory courses and vocational education. Written information about the study was provided to students and their guardians. The focus group interviews were conducted from May to October 2021 at school, with the first and second authors taking turns being moderators and observers. The moderator and observer, both females, had several years of experience working as registered nurses. The third and fourth authors, pediatric nurses with doctoral degrees, had extensive experience in focus group interviews with children and adolescents. They provided training and supervision to the first and second authors. Before the interviews began, the students were informed that the interview would be recorded on an audio file; the student’s participated voluntarily, and written informed consent was obtained. The researchers and the respondents had no relationship before study commencement. 

### 2.2. Setting and Participants

A total of 41 adolescents (32 females and nine males) were interviewed in nine focus groups (Table 1). During the period in which the interviews were conducted, the schools had returned to on-site education after a period of distance education because of the COVID-19 pandemic. The interviews were conducted in the schools’ undisturbed rooms and lasted 30–60 min. The adolescents sat around a common table where everyone was included in the conversations. By the participants themselves choosing when and how actively they participate in the conversation, it becomes more voluntary to choose to what extent he or she is comfortable participating. The size of focus groups can vary, but it is advantageous to use smaller focus groups as more space to speak is given to all participants [26]. This study held focus group interviews with three to six participants. The focus group interviews were based on a semi-structured interview guide with open-ended questions.

The interview guide has been used previously among 13–15-year-old adolescents [5] and 10–12-year-old children [4]. Examples of questions are “What do you think when you hear the word stress?” and “Can you describe situations when stress has been good/bad?” The research project on stress in children and adolescents started before the pandemic. Initially, therefore, the purpose was stress in general, but when the COVID-19 pandemic arrived in 2020, it became part of the young people’s everyday life, which is why that issue was also discussed in the focus group interviews. A question was added in the interview guide after 2020: “What do you do to feel good? Has that changed during the pandemic?” Using semi-structured questions gives the participants space to bring up their thoughts and is suitable for exploratory studies [26]. The first focus group interview was considered a pilot interview. After the first focus group interview, the first, second, and fourth authors met and discussed the interview, and found that the interview guide worked well and decided to include the pilot interview in the analysis. 

### 2.3. Analysis

As an analysis method, Hsieh and Shannon’s [27] conventional qualitative content analysis was used to process the material. All interviews were initially transcribed by the first and second authors. Then, the transcripts were reviewed to ensure that the content was perceived similarly and that nothing was missed in writing. For categories to develop from the text, the transcripts were read through repeatedly, which allows for an inductive development of categories [27]. The interviews were entirely analyzed and could be broken down into meaning units. Sentences and passages in the transcript concerning stress were marked and transferred to a new document to exclude redundant material. This was done primarily by the first and second author, and then sentences and paragraphs were compared to ensure that the same content was considered relevant. In the next step, words or shorter phrases that concerned experiences of stress were marked and coded in the margin. All codes that emerged were compiled in a new document where the codes were reviewed, and duplicates were combined; further review compiled the codes into subcategories together with the last author. In the last step, all four authors discussed the subcategories that guided the overall areas that emerged and then formed the final categories. All authors discussed the analysis until a consensus was obtained. 

## 3. Results

Adolescents’ experiences of stress were mainly linked to school and school assignments; all adolescents had an experience of experiencing stress at school. There were also stressful situations in leisure time and relationships (romantic, non-romantic, and family relationships). Stress affected adolescents both mentally and physically and was perceived as negative. The adolescents said that moderate stress increased performance and was perceived as necessary and positive. The adolescents had several strategies to counteract or prevent stress. Five categories were identified from the results: (1) School-related stress, (2) Stress in relationships and leisure, (3) How stress manifests itself, (4) Stress can increase performance, and (5) Experiences of counteracting stress. Each of these are addressed below.

### 3.1. School-Related Stress

Most adolescents mainly experience stress related to school. At school, imminent stress with several tasks simultaneously, time pressure, being behind with school tasks, and high-performance requirements on oneself were cited as causes of stress. The adolescents had the experience of postponing work tasks, which increased stress and made it more difficult to get to grips with the tasks. It took most of the adolescents’ time and energy to perform well in school, and under high stress, the ability to concentrate could decrease, which created a vicious circle. Most adolescents experienced that the stress disappeared or decreased during holidays and vacations, and stress was mostly associated with the school.

*“Yes, I myself am not so stressed in my spare time. But if that were the case, it would be because of the school. I feel stressed that I am behind in something” Girl, Focus Group 2*

*“For me, it will be that I do not know where to start, so if there is a lot of schoolwork, it will be where should I start. And then it can happen that you don’t bother about anything in the end because you do not know where to start” Girl, Focus Group 5*

Most adolescents stated that school, when they were 13–15 years old, was more stressful than now in upper secondary school. Stress over grades, getting into the chosen upper secondary school, and several subjects in junior high school created difficulties in prioritizing and performing well. Several adolescents experienced pressure from the school and the teachers.

*“You were a little pressured because you would build for your future and improve the grades so that you would be a competent student with the best grades and the best job… it was as if they presented it as if you do not get average or top marks so that you do not manage, but you actually do...” Girl, Focus Group 9*

*“So really, the upper secondary school does not stress me so much, because you often do everything in the classes, at least in our class but before, in school when you were 13–15 years old, then it was excessively stressful because then it was like all the subjects in one day and you have long classes, you do not have time to finish everything in 40 min in one class”. Girl, Focus Group 6*

Shorter classes and several classes in different subjects made it more difficult to focus on one subject at a time. Some adolescents stated that the teachers in the various subjects had no or little communication about the planning of assignments, and having several assignments and tests during the same period caused high stress. Most adolescents experienced less stress in upper secondary school. The stress of getting into upper secondary school disappeared; they were given more freedom, had fewer subjects, and had more intensive learning periods, which facilitated school time and reduced stress. Some adolescents continued to stress when thinking about the future.

*“You have a test that you do not pass; what happens next? What should I do if I do not pass the subject, for example, when I have to do it again?” Boy, Focus Group 1*

The adolescents had mixed experiences of school-related stress during the pandemic. Most adolescents reported negative experiences. Distance education affected their studies, and some students fell behind with tasks, which increased stress. It was more difficult to ask for help during distance education. Some had problems focusing on school assignments; it was also difficult to distinguish between school and home environments. Some felt that they concentrated better at home than at school and that schoolwork became more efficient.

*“It is probably mostly the school [that causes stress] that when you have always had distance education [because of the COVID-19 pandemic], you do not have a teacher next to you who you can ask for help all the time. I got stressed because I was so behind. They had not provided the enough help I needed”. Girl, Focus Group 2*

*“I think it’s harder. When we had distance education, it was much harder to get things done when you were at home and working, because it is like not the same, type, it feels like when you are sick, then you are at home and then work, but usually not. When you are at school then you work, you are at home and have to work, it becomes more unfocused, it’s like you just study and do not take it as seriously as you do at school”. Girl, Focus group 6*

### 3.2. Stress in Relationships and Leisure

The adolescents experienced stress over relationships. Several adolescents expressed stress as a concern and uncertainty about the future. However, insecurity in relationships with others is also founded on a fear of losing a partner or friend. Some students experienced stress about finances and family situations and found that they went into a more adult role during upper secondary school.

*“Relationships are more demanding. Because in school there is someone who says how to do and what to do, there you get like instructions. In relationships, it’s more, so there are two, it’s a collaboration all the time. And you have to know what the other wants and what you want and what to do and what the other thinks when it does not tell what it thinks and maybe things like that”. Girl, Focus Group 9*

Friendships outside the family become more important in upper secondary school, and conflicts between friends cause stress, being forced to choose between friends and the fear of losing a friend. Insecurity, jealousy, and fear of being replaced are causes of stress in relationships that some participants raised. Thoughts about how others perceive them in school were also a stress factor for the students; the adolescents gave examples of moderate and high stress in social situations. Some adolescents experienced a difference during the pandemic when the school switched to distance education, and they were more isolated from social contacts. The majority of adolescents experienced social distancing as negative. However, there were also advantages as they were less stressed about appearance and how others perceived them. Social media affects adolescents. Some felt more sensitive to influence and chose to refrain from certain social applications, while others were less affected. It also emerged that family relationships could affect how one experiences stress; some adolescents experienced high stress related to an insecure home situation.

*“Then it will be a stress if you are not chosen, then you think, what did I do wrong? Why am I not as good as that person? And then the thoughts start to roll even more” Girl, Focus Group 2*

*“It feels like now, when you get out to people, then it feels like you think about what you do all the time. Because it has been a period [during the COVID-19 pandemic] where you have been trapped in yourself as well, and you kind of stop caring about what I dressed in and stuff like that, but now it’s kind, important it feels like. It feels like you become, you get judged if you do not do the right thing. Because you’ve been away from people for so long”. Girl, focus group 8*

### 3.3. How Stress Manifests Itself

Most adolescents expressed that today they did not experience any prolonged stress or high stress in everyday life but that stress was manageable and transient. However, all adolescents had previous experiences of stress and were able to describe several psychosomatic symptoms that they have experienced during periods with high-stress levels or stress for a long period. The adolescents described problems such as headaches, depressive symptoms, body aches, and difficulty sleeping. When the students answered what they thought when they heard the word stress, lumps in the stomach, anxiety, and negative thoughts could be described. All adolescents who described stress reported negative associations with an unpleasant feeling in the body. Several adolescents had experiences of memory difficulties, difficulty concentrating, and becoming passive with a feeling that the body was shutting down. Some students experienced panic attacks, rapid heartbeats, and daily stress.

*“...I notice myself when I’m stressed, it usually is, I’m kind of like when you’re restless. You know I can not sit still, when I am stressed, when I am very stressed I, there are too many things going on in my head, so I can not focus on one thing. If someone talks to me when I’m stressed, I just get a little like he said he turns himself off, so I’m a little so ah okay, I don’t care in what I do not listen, I just close my ears, so focus in my head it’s going on a hundred things at once”. Boy, Focus Group 7*

*“I kind of start sweating and kind of my head goes up in laps and I kind of hyperventilate-and get red, so hot and red in the face. I get stressed out a lot, ass all the time”. Girl, Focus Group 4*

Stress was perceived by adolescents to be subjective and to affect people to varying degrees. Most adolescents could give examples of how stress manifests itself in others; for example, they seem to be tense, walk fast, have changed body language, sweat, or are forgetful. Stressed people could be perceived as easily irritated or angry but depressed or have a change in tone of voice. The adolescents experienced that it is easier to tell whether people they know are stressed than strangers. Some adolescents reflected that stress could change people’s behavior; those who talk or eat often talked or ate less when stressed and vice versa.

*“Yes, but the fact that people are easily irritated, I often think, is that you are stressed, that you, yes, it is very easy to make them angry”. Girl, Focus Group 8*

### 3.4. Stress Can Increase Performance

Most adolescents thought mainly of negative experiences of stress; with more reflection, most could reflect on how stress motivated them to finish tasks or arrive on time. Stress was experienced as increasing performance in school and became a driving force for studying before exams and submitting assignments on time. When tasks were finished, the stress was often released and described as a pleasant feeling. The adolescents experienced that moderate stress could increase motivation and improve their performance. One student gave examples of how stress could be motivating, helping to get in better shape or reach personal goals. Some adolescents felt that stress in a sports context was positive as it improved performance and, in turn, is appreciated by teammates. Some adolescents reflected that stress was a strain in life needed to build self-esteem and self-confidence. All adolescents have experience that stress made them arrive on time for school and other things.

*“I think that stress can be quite good when it comes to arriving on time and such... And you kind of, yes, you get started with the work or type for the test, and in that case you might have arrived late by then. I actually think that stress is pretty good, because it fights you so that you do not just “I can not seem to cope””. Girl, Focus Group 3*

*“I have a goal in life for example if I want better... better body maybe, if I want to lose weight or I want to gain weight... I usually feel that eh stress is good, stress is useful as motivation that now and I know that deep down I know I have to do it no matter how stressful it gets I have to do this because I want it myself”. Boy, Focus Group 7*

The adolescents stated that moderate and transient stress is positive. However, if you experience high stress for a long period, it affects your health and mental state.

*“Stress is good to a certain level. If it becomes too much, it becomes negative, but as a type as in such contexts, it is quite low stress anyway, so the body can still cope with it and do something good out of it, but if it becomes too much, yes, it becomes, everything gets worse”. Girl, Focus Group 2*

### 3.5. Experiences of Counteracting Stress

The adolescents described what they usually do to counteract stress. This included activities or chores they did to feel good or help and support from others to counteract stress. The adolescents also reflected on destructive behaviors in dealing with mental health problems. The adolescents experienced that stress became more manageable by creating a good structure and planning. Most adolescents felt the need to be able to prioritize well between tasks and activities. Some adolescents were able to get help from their parents to prioritize and structure tasks so that schoolwork would be more manageable, and the stress only be released when the tasks were completed. The stress then went from high to moderate intensity. The adolescents could sometimes choose to postpone tasks and spend time on things like hanging out with animals, resting, playing computer games, exercising, taking time off or hanging out with friends, listening to music, and working on car mechanics, which helped them unwind to deal with stress. Some adolescents felt that the teachers gave time in the classes to catch up on homework and assignments, which meant that the stress in their free time was not affected by schoolwork.

*“Planning things up. Write down everything, all the homework you have and when they should be handed in and try to do most things at school, so you do not have to do it at home”. Girl, Focus Group 6*

The participants reported that how you are as a person could impact how you are affected by stress, which was clear when they compared themselves with others and how they were affected by stress.

*“Yes, but for example, if we two get the same problem, for me it’s big for him it’s small it depends on how eh it’s environment he grew up [in]...” Boy, Focus Group 7*

The adolescents reflected on how prolonged stress could lead to reduced mental well-being and how they handled stress. Some adolescents reported that they had experiences of themselves or others in their vicinity developing destructive behaviors when they had reduced mental well-being. Destructive behaviors like experimenting with sex, drugs, alcohol, and smoking or focusing on weight loss or harming oneself. One young man said that driving dangerously fast helped him cope with stress:

*“There are some negative things that help against stress extremely much. For me, speeds, I love to chase speeds, and that.. to go, when you drive for over 200 km/h you do not think of anything else you think of “oh damn how fast it goes like”. You are somewhere else for everything just whizzes by; you’re there then”. Boy, Focus Group 7*

*“Well, I usually sleep a lot, I usually eat, and for many years I have had many different types of self-harming behaviors. Everything from the fact that I have had gambling addictions to the fact that I have had alcohol abuse to the fact that I have had similar sex addictions...” Boy, Focus Group 7*

## 4. Discussion

The most prominent finding was that adolescents linked stress to school work. Factors outside of school also create stress for adolescents, especially relationships with others. Although Sweden chose a milder lockdown than many other countries during the COVID-19 pandemic, distance education during the COVID-19 pandemic increased the experience of stress and the majority of adolescents experienced social distancing during COVID-19 as negative. The adolescents felt that they could better manage stress now during upper secondary school (age 16–19) than during junior high school (age 13–15). It seems that adolescents’ ability to cope with stress changed as they gained maturity and experience managing stress. 

Most experiences of stress were related to schoolwork. Previous experiences of stress were from junior high school, when the students reported that stress was difficult to manage. This is in line with most other studies showing that school-related work was the main cause of stress even at younger ages [5,7,9,10]. In upper secondary school, the adolescents experienced that the pressure from school decreased compared to junior high school. Fewer subjects, longer class time, and less pressure from the environment could contribute to increased comprehension among adolescents. Comprehensibility in Antonovsky’s [16] theory is when the outside world is perceived as clear, orderly, and structured, and then conditions are beneficial to handle stressful situations [16]. In upper secondary school, stress occurred at high workloads but was perceived to be more manageable.

Manageability is when you create control in situations and can solve problems using your resources, including your abilities or people close to you [16]. The adolescents experienced that, in upper secondary school, it is easier to structure their schoolwork in combination with leisure interests and other areas of responsibility, which could be because age and experience make it easier to handle stress. The adolescents describe that they experience greater freedom and may take more responsibility in upper secondary school, which can be explained by the fact that studies at the upper secondary school level are voluntary and that students do not have compulsory schooling at upper secondary school. Studies at the upper secondary school level give adolescents the opportunity to choose programs based on their interests, which may explain why adolescents feel that their studies are meaningful, increasing their motivation. Meaningfulness is the degree to which one is motivated to solve problems. It takes a willingness to face difficulties and invest energy in getting through what causes stress [16]. A study by Garcia-Moya et al. [18] showed that a supportive school contributed to students experiencing a higher sense of coherence, which was important in managing stress. With a strong sense of coherence, the adolescents handled demands from school without being stressed. The stress threshold was lower with a low sense of coherence, and schoolwork became less comprehensible. This, in turn, caused a decrease in self-confidence, and that schoolwork became less meaningful [18].

Other factors were stress in relationships with others. Family relationships and friendships are important to adolescents, and changes in relationships with others are more significant. The responsibility to nurture relationships is greater, and the fear of losing a friend or partner is a stress factor. Likewise, thoughts and uncertainty about how other people perceive them contribute to stress. This is in line with Camara et al.’s [28] study that reports that adolescents experienced concern about being judged by others. Friendships can act as a stress factor when there is fear of being rejected, as adolescents need to be part of a social context [28]. In the focus group interviews, some adolescents said that social media is a stress factor, especially when they compare themselves with others. This creates performance anxiety and stress about being worse than others. That adolescents compare themselves with others is a normal part of social development and can contribute to self-development, but it has also been shown to be a stress factor in social media [29]. 

In the focus group interviews, the adolescents gave several examples of how they have developed strategies to counteract stress, both in ways that they found to be positive and healthy, but also in negative ways, such as substance use or driving too fast. Positive examples were taking a walk, hanging out with animals or friends, or taking time for themselves to unwind. This is in line with a study by Wilhsson et al. [30], who examined strategies in adolescents to counteract school-related stress. Spending time with family and friends could give the students more energy and greater ability to handle demands from school. There was also a difference in how boys and girls handled stress [30]. Some adolescents stated that stress could be managed by seeking help from their parents, structuring schoolwork, and receiving support in dealing with particular situations. A study by Garcia-Moya et al. [19] emphasized the importance of the family in increasing the sense of coherence, which provided greater resilience. Adolescents experienced that relationships with parents and friends could cause stress, but at the same time, support helped them deal with stress in different situations [28].

### Strengths and Limitations

The strengths of the study are the relatively high number of participants and the variety of views that were expressed. Another strength is that the analysis was conducted jointly with the four researchers. Study limitations are the question of transferability since the study was conducted in two municipalities in southern Sweden. Although the upper secondary school had distance education during part of the COVID-19 pandemic, Sweden had relatively less strict lockdowns than other countries [22]. It is therefore not obvious that the results could be generalized into other contexts. The method with focus group interviews has both pros and cons. The benefits of a focus group interview are that it allows a variety of views to flourish, and for adolescents, it could also be beneficial to be interviewed together with peers as peers are central in this age group [26]. However, individual interviews could be advantageous if the respondents do not want to share their thoughts in a group and to achieve a greater depth in the interviews. In this study, the focus group interviews went well, and the interviews provided rich data material. The research aim was initially to investigate stress in this age-group in general, but the COVID-19 pandemic arrived before the focus group interviews started, and therefore stress during the pandemic is investigated. We suggest that a new study on stress among adolescents should be done in the future when the pandemic is over as well. 

## 5. Conclusions

Our results confirm that stress was a well-known experience among students aged 16–19 and that stress was primarily governed by the school’s design. Distance education during the COVID-19 pandemic increased the experience of stress. High stress harms adolescents and reduces school performance, while moderate stress could contribute to being on time, completing tasks, and motivating people to achieve goals. The adolescents had negative experiences of stress associated with school in earlier years. Students experienced mostly manageable stress in upper secondary school, which helped them perform better in school. 

This study sheds light on adolescents’ experiences of stress and finds that stress during school was difficult to manage, especially during distance education, due to the COVID-19 pandemic. Both healthy and unhealthy coping behaviors were described to counteract stress. More research is needed on how school health services can improve adolescents’ coping with stress in regard to their age and development to become more manageable, comprehensible, and meaningful. A school that increases the sense of coherence, in turn, fosters better resilience to stress among its students.

## Figures and Tables

**Table 1:** Description of focus groups.

Focus Group	School	Females	Males	Total
1	A	4	1	5
2	B	4	0	4
3	B	4	0	4
4	B	3	1	4
5	B	3	0	3
6	A	4	2	6
7	A	1	4	5
8	B	5	0	5
9	B	4	1	5
Total:		32	9	41

## Data Availability

The data are not publicly available due to ethical reasons.
